# Magnetic phase separation in microgravity

**DOI:** 10.1038/s41526-022-00212-9

**Published:** 2022-08-08

**Authors:** Álvaro Romero-Calvo, Ömer Akay, Hanspeter Schaub, Katharina Brinkert

**Affiliations:** 1 Department of Aerospace Engineering Sciences, University of Colorado, Boulder, CO 80303 USA; 2 Department of Physics, Freie Universität Berlin, 14195 Berlin, Germany; 3 ZARM—Center of Applied Space Technology and Microgravity, University of Bremen, 28359 Bremen, Germany; 4 Department of Chemistry, University of Warwick, CV4 7AL Coventry, UK

**Keywords:** Aerospace engineering, Fluid dynamics, Applied physics, Mechanical engineering, Chemical engineering

## Abstract

The absence of strong buoyancy forces severely complicates the management of multiphase flows in microgravity. Different types of space systems, ranging from in-space propulsion to life support, are negatively impacted by this effect. Multiple approaches have been developed to achieve phase separation in microgravity, whereas they usually lack the robustness, efficiency, or stability that is desirable in most applications. Complementary to existing methods, the use of magnetic polarization has been recently proposed to passively induce phase separation in electrolytic cells and other two-phase flow devices. This article illustrates the dia- and paramagnetic phase separation mechanism on MilliQ water, an aqueous MnSO_4_ solution, lysogeny broth, and olive oil using air bubbles in a series of drop tower experiments. Expressions for the magnetic terminal bubble velocity are derived and validated and several wall–bubble and multi-bubble magnetic interactions are reported. Ultimately, the analysis demonstrates the feasibility of the dia- and paramagnetic phase separation approach, providing a key advancement for the development of future space systems.

## Introduction

Human space exploration is presented with multiple challenges, to the fore, the absence of buoyancy forces in orbit. This has severe complications for phase separation in microgravity environments, which is, however, a crucial process for a wide variety of space technologies. These include propellant management devices, heat transfer, and life support systems comprising the production of oxygen, fuels, and other chemicals as well as the removal of carbon dioxide from cabin air and the recycling of wastewater, among many others.

Numerous phase separation methods have been developed for microgravity conditions. Centrifuges^1,2^, forced vortical flows^3,4^, rocket firing^5,6^, membranes^7,8^, and surface-tension-based technologies^9,10^, which include wedge geometries^11–14^, springs^15^, eccentric annuli^16^, microfluidic channels^17^, or porous substrates^18,19^, among others, are the most traditional solutions. As an alternative, the use of electrohydrodynamic forces has been studied since the early 1960s^20^ and successfully tested for boiling^21–23^, two-phase flow management^24,25^, and conduction pumping^26^ applications. Hydroacoustic forces arising from the application of ultrasonic standing waves^27^ have been used to enhance a wide variety of terrestrial processes^28^ and are also proposed to control bubbly flows in propellant tanks^29,30^ and life support systems^31^. Small amplitude vibrations can also be employed to manage multiphase flows and induce phase separation in microgravity^32^ by selecting viscoequilibrium configurations^33^ or exploiting frozen wave instabilities^34^. These approaches present unique characteristics that affect aspects like their operational lifespan, reliability, performance, and intrusiveness^31^.

Complementary to the aforementioned methods, the inherent dia- and paramagnetic properties of liquids can be employed for passive phase separation^35^. Inhomogeneous magnetic fields induce a weak volume force in continuous media^36^ that, due to the differential magnetic properties between phases, results in a net buoyancy effect. This phenomenon is known as *magnetic buoyancy* and has been applied to terrestrial boiling experiments with ferrofluids^37,38^. Previous works on low-gravity magnetohydrodynamics have explored the diamagnetic manipulation of air bubbles in water^39,40^, the positioning of diamagnetic materials^41^, air-water separation^42^, protein crystal growth^43^, magnetic-positive positioning^44,45^, magnetic liquid sloshing^46,47^, and combustion enhancement^40^, among others. The application of Lorentz’s force on liquid electrolytes has also been studied as a way to enhance hydrogen production^48–50^. The use of magnetic buoyancy in phase separation under microgravity conditions remains, however, largely unexplored.

The discovery of diamagnetism dates back to 1778 when A. Brugmans reported the diamagnetic effect on bismuth^51^. In 1845, M. Faraday demonstrated that magnetism is a universal property of matter and carried out the first thorough study of the phenomenon, classifying different materials as “diamagnetic” and “paramagnetic”^52^. From a macroscopic perspective, diamagnetic and paramagnetic substances are respectively repelled and attracted by magnetic dipoles by means of the Kelvin body force^53^

1 $${{{\bf{f}}}}={\mu }_{0}M\nabla H,$$

where *μ*_0_ is the magnetic permeability of free space, and *M* and *H* are the modules of the magnetization (**M**) and magnetic (**H**) fields, respectively. The volume magnetic susceptibility of a soft magnetic material, *χ*^vol^, is defined through **M** = *χ*^vol^**H**, and its sign determines whether a substance is diamagnetic or paramagnetic. The magnetic polarization force on natural liquids is so weak that its effects on Earth are usually negligible. However, in a microgravity environment this weak interaction leads to a magnetic buoyancy effect that can be exploited to induce phase separation^35^.

Herein, this paper reports the first comprehensive study of magnetically induced buoyancy in microgravity environments generated for 4.7 s at the drop tower of the *Center for Applied Space Technology and Microgravity* (ZARM). The artificially created buoyancy force is utilized to direct air gas bubbles on specific trajectories through dia- and paramagnetic solutions. The results demonstrate that the inherent magnetic properties of these substances are sufficient to allow the collection and coalescence of gas bubbles at distinct locations of the experiment vessel, providing a proof of concept that the development of microgravity magnetic phase separators could lead to reliable and lightweight space systems.

## Results and discussion

### Overview

Gas bubbles are the elemental multiphase flow unit and represent the main focus of this work. The application of dia- and paramagnetic buoyancy to bubble management in microgravity is subsequently demonstrated with liquids of technical interest. Five 4.7 s microgravity experiments, listed in Table 1, are performed at ZARM’s drop tower. During each drop, bubbles are simultaneously injected inside three syringes filled with the same carrier liquid. The first two drops employ MilliQ water, whose properties are well-characterized. This motivates the adoption of these experiments in the validation of theoretical results. An aqueous 0.5 M manganese (II) sulfate solution (MnSO_4_ ⋅ H_2_O) is employed in the third drop to demonstrate the paramagnetic buoyancy effect. The fourth drop employs Lysogeny Broth (LB) medium (*Miller*), which is widely used in biological experiments on the International Space Station for the growth of bacteria^54^, to demonstrate how the diamagnetic effect can be used to induce phase separation in such applications. Finally, extra-virgin olive oil is tested to exemplify how phase separation takes place in a complex organic solution. Two syringes are exposed to the inhomogeneous magnetic field generated by a magnet located at the left (L) and right (R) of the sample volume, while the third is used as a non-magnetic control (C). The magnetic environment is designed to induce a lateral buoyancy effect on the liquid and is fully characterized in sec. “Wall–bubble interactions”. From now on, each video recording will be labeled as *N*-*X*, where *N* denotes the sample type (L, R, or C) and *X* the drop number (01 to 05).

**Table 1:** Liquids employed on each drop experiment.

ID	Liquid	Classification
01	MilliQ Water	Diamagnetic
02	MilliQ Water	Diamagnetic
03	0.5M MnSO_4_⋅H_2_O(aq)	Paramagnetic
04	LB medium	Diamagnetic
05	Extra-virgin olive oil	Diamagnetic

**Fig. 1:**
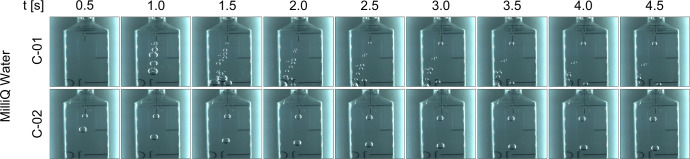
Non-magnetic control (C) experiments for MilliQ water in microgravity. The injection and displacement of air bubbles are shown as a function of time.

Control video samples are shown in Fig. 1 for the first two drops with MilliQ water. A wide range of bubble diameters is generated due to the varying pressure conditions and unsteady nature of the experiment. This feature will be useful to understand how different bubble diameters behave in the presence of the magnetic field. The injection of gas in the syringe leads to a downwards movement that is mostly damped after ~3 s. A slight lateral deviation of the flow is occasionally observed due to the small irregularities in the tip of the injector. Similar behaviors are repeated in the other three control videos, which have been omitted for clarity. The interested reader is referred to the Supplementary Materials to access these additional recordings.

**Fig. 2:**
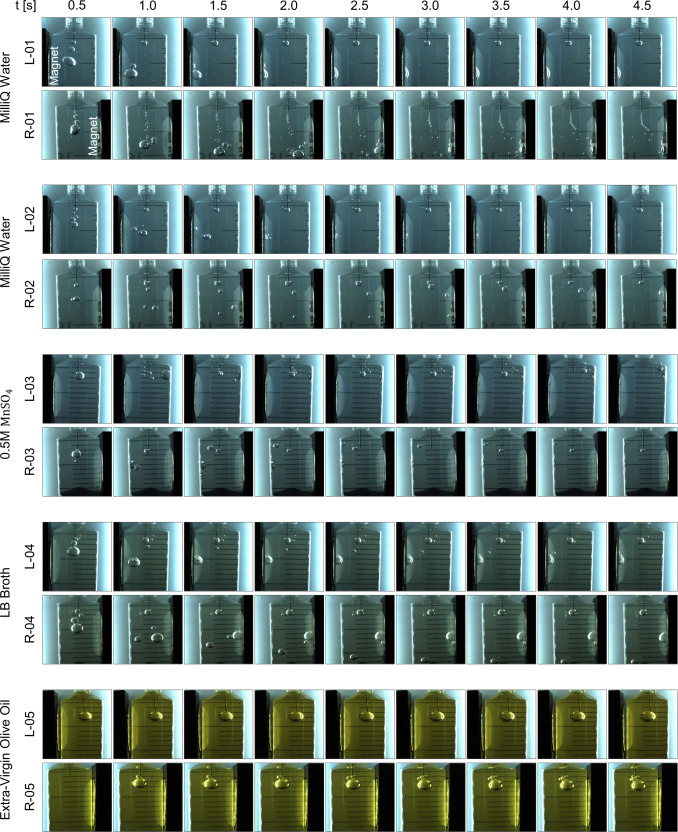
Overview of dia- and paramagnetic results for left (L) and right (R) magnetic configurations and the five drop experiments. The injection and displacement of air bubbles are shown as a function of time.

Magnetic results for the five drops under study are depicted in Fig. 2. The bubbles are collected by the magnets in all diamagnetic cases (01, 02, 04, and 05), while they are pushed away in the paramagnetic scenario (03). Complex mixtures like LB Broth and olive oil are significantly affected by the magnetic force. In the second case, the effect is less noticeable (but still visible) due to the higher viscosity of the liquid, which increases the drag acting on the bubble. This is an example of how all liquids are subject to magnetic polarization forces and can therefore be employed to induce phase separation in microgravity environments. The determination of such response, represented by the magnetic susceptibility, is relatively straightforward for simple solutions^35,55^. Complex mixtures, on the contrary, need to be characterized with magnetometers.

The experiments reveal key information about the dynamics of bubbles subject to inhomogeneous magnetic fields in microgravity, as discussed in sec. “Terminal velocity”. Several additional interactions of particular technical interest are also described in sec. “Wall–bubble interactions” and sec. “Bubble–bubble interactions”.

### Terminal velocity

The determination of the terminal (or steady-state) bubble velocity under the action of gravity has historically raised significant attention due to its importance for a wide range of industrial applications. The problem is severely complicated by factors like the bubble radius, shape, and formation method as well as the liquid purity, viscosity, temperature, and pressure^56^. In spite of this inherent complexity, three distinct dynamic regimes can be observed: viscosity-dominated, surface-tension-dominated, and inertia-dominated^57–59^. The dynamic regime of a given bubble is determined by the balance between fundamental forces. The Weber number

2 $${{{\rm{We}}}}=\frac{{\rho }_{{{{\rm{l}}}}}{V}^{2}(2R)}{\sigma }$$

reflects the ratio between inertial and surface-tension-induced pressures, with *R* and *V* being the bubble radius and velocity, *ρ*_l_ the liquid density, and *σ* the coefficient of surface tension. This ratio is much smaller than one for the radii and velocities covered in this work, indicating that bubbles remain almost perfectly spherical. On the other hand, the Reynolds number

3 $${{{\rm{Re}}}}=\frac{{\rho }_{{{{\rm{l}}}}}V(2R)}{\eta },$$

where *η* is the dynamic viscosity of the liquid, describes the ratio of inertial to viscous forces and is kept below 50 in this work, as shown later on. The combination of low $\rm{We}$ and moderate ${{{\rm{Re}}}}$ numbers results in spherical bubbles with no-zigzag motions. Therefore, due to the weakness of the diamagnetic force and the overwhelming role of surface tension, the case of a free-floating air bubble subject to the influence of a magnet in microgravity falls within the viscosity-dominated bubble displacement regime. In terms of fluid motion, the flow remains attached to the bubble until ${{{\rm{Re}}}}\approx 20$, where it is separated at the rear stagnation point leading to a steady wake region that remains stable until ${{{\rm{Re}}}}\approx 130$^57^.

In the dynamic regime of interest, the movement of a spherical bubble in a liquid is described by the balance

4 $$m^{\prime} \frac{{{{{\rm{d}}}}}^{2}{{{\bf{x}}}}}{{{{\rm{d}}}}{t}^{2}}={{{{\bf{F}}}}}_{{{{\rm{m}}}}}^{\,{{\mathrm{eff}}}\,}+{{{{\bf{F}}}}}_{{{{\rm{d}}}}}+{{{{\bf{F}}}}}_{{{{\rm{h}}}}},$$

with $m^{\prime} =(4/3)\pi {R}^{3}({\rho }_{{{{\rm{g}}}}}+0.5{\rho }_{{{{\rm{l}}}}})$ being the virtual mass (that accounts for the surrounding fluid accelerated by the bubble^60^), *ρ*_g_ the gas density, **x** the position of the bubble, ${{{{\bf{F}}}}}_{{{{\rm{m}}}}}^{\,{{\mathrm{eff}}}\,}$ the magnetic polarization force, **F**_d_ the viscous drag, and **F**_h_ the history (or Basset) force^61^. The total magnetic polarization force acting on the bubble is^35^

5 $${{{{\bf{F}}}}}_{{{{\rm{m}}}}}^{\,{{\mathrm{eff}}}\,}\approx \frac{2}{3}\pi {R}^{3}{\mu }_{0}{{{\rm{{{\Delta }}}}}}{\chi }^{{{\mathrm{vol}}}}\nabla {H}_{0}^{2},$$

where ${{{\rm{{{\Delta }}}}}}{\chi }^{{{\mathrm{vol}}}}={\chi }_{{{{\rm{b}}}}}^{\,{{\mathrm{vol}}}\,}-{\chi }_{{{{\rm{e}}}}}^{\,{{\mathrm{vol}}}\,}$ is the differential magnetic susceptibility between the gas and the surrounding medium. This expression is valid for small bubbles and low-susceptibility gases and liquids, for which the external magnetic field module in the absence of magnetized samples, *H*_0_, is used as an approximation of *H*.

When a rigid body and Stokes flows (${{{\rm{Re}}}} \,<\, 1$) are considered, the drag force can be modeled with the Stokes law^62^

6 $${{{{\bf{F}}}}}_{{{{\rm{d}}}}}=-6\pi R\eta ({{{\rm{d}}}}{{{\bf{x}}}}/{{{\rm{d}}}}t).$$

This expression is appropriate in technical applications where the liquid is exposed to impurities and the so-called “Marangoni” effect blocks the bubble surface movement. In particular, water is extremely sensitive to surface contamination^63,64^, and even the contact with the atmosphere can immobilize its surface^65^. Pure liquids exhibit a mobile interface that promotes the circulation of air inside the bubble. In these cases, the Hadamard-Rybczynski drag force^66,67^, validated on Earth using ultra-clean systems^65,68^, should be employed instead. Intermediate formulations with partially mobile surfaces have also been proposed^69^.

However, the Stokes law is not valid for ${{{\rm{Re}}}} \,>\, 1$, and a different formulation is thus required in this regime. Most results are based on experimental or numerical works where the drag force

7 $${{{{\bf{F}}}}}_{{{{\rm{d}}}}}=-\frac{1}{2}{\rho }_{{{{\rm{l}}}}}{V}^{2}A{C}_{{{{\rm{D}}}}}\frac{{{{\rm{d}}}}{{{\bf{x}}}}/{{{\rm{d}}}}t}{\parallel {{{\rm{d}}}}{{{\bf{x}}}}/{{{\rm{d}}}}t\parallel }$$

is defined by means of a drag coefficient *C*_D_, with *A* = *πR*^2^ being the reference area of the spherical bubble. Numerous correlations have been proposed for the range ${{{\rm{Re}}}}\in [0.01,100]$, one of the simplest being given by Rumpf

8 $${C}_{{{{\rm{D}}}}}=\kappa +\frac{24}{{{{\rm{Re}}}}},$$

where *κ* = 2 for ${{{\rm{Re}}}}\in [0.01,10]$ (±5% error) and *κ* = 1 for ${{{\rm{Re}}}}\in [10,100]$ (±20% error)^57^. Although more accurate formulations have been derived^57,70^, this particular one simplifies the derivation of analytical closed-form results.

The magnetic terminal velocity for a Stokes flow is obtained after assuming a steady-state behavior in Eq. (4) and considering Eq. (6), resulting in^35^

9 $${v}_{{{{\rm{t}}}}}\approx \frac{{\mu }_{0}{R}^{2}}{9\eta }{{{\rm{{{\Delta }}}}}}{\chi }^{{{\mathrm{vol}}}}| | \nabla {H}_{0}^{2}| | ,\,\,\,{{{\rm{Re}}}}\,<\, 1.$$

For ${{{\rm{Re}}}} \,>\, 1$ the application of Eq. (7) results in the terminal velocity

10 $${v}_{{{{\rm{t}}}}}\approx \frac{-9\eta +\sqrt{3}\sqrt{\kappa {\mu }_{0}{\rho }_{{{{\rm{l}}}}}{R}^{3}{{{\rm{{{\Delta }}}}}}{\chi }^{{{\mathrm{vol}}}}| | \nabla {H}_{0}^{2}| | +27{\eta }^{2}}}{(3/2)R\kappa {\rho }_{{{{\rm{l}}}}}},\,\,\,{{{\rm{Re}}}}\in [0.01,100],$$

which can be useful for first-order bubble velocity estimations. It is important to emphasize that both Eq. (9) and Eq. (10) are only valid for steady-state systems. In this work, the inhomogeneity of the magnetic force and the short duration of the drop tower experiments prevent bubbles from reaching their terminal velocity. Still, this value can be employed as an upper-speed limit, hence becoming a powerful characterization metric.

In order to evaluate the performance of Eqs. (9) and (10), the radius, maximum and minimum speed, maximum and minimum Reynolds number, and interaction history of 25 air bubbles in water are reported in Table 2 after being analyzed with the tracking algorithm described in sec. “Bubble tracking algorithm”. The analysis focuses on the *x* (“horizontal”) vector components, where magnetic effects are dominant and the injection velocity is negligible. The maximum horizontal bubble velocity is 15.9 mm s^−1^, which corresponds to ${{{\rm{Re}}}}=45.1$, while the minimum is just 0.1 mm s^−1^. As a consequence of Eqs. (9) and (10), larger bubbles generally have higher maximum velocities. From a technical perspective, this implies that the diamagnetic phase separator is more effective with large bubbles. Smaller bubbles, on the contrary, are slower but show a higher velocity scattering due to the bubble interaction effects described in sec. “Bubble–bubble interactions”.

**Table 2:** Bubbles tracked during 4.7 s of free fall. A unique label is assigned to each bubble for the L-01, L-02, R-01, and R-02 experiments.

ID	*R* [mm]	$-{v}_{x,\min }$ [mm s^−1^]	$-{v}_{x,\max }$ [mm s^−1^]	$R{e}_{\min }$ [-]	$R{e}_{\max }$ [-]	Interacts?
L-01-01	2.55	4.7	15.9	13.4	45.1	Yes
L-01-02	1.26	7.8	12.1	10.9	16.9	Yes
L-01-03	0.37	1.6	2.8	0.6	1.13	No
L-02-01	1.45	–1.5	14.2	0	22.7	Yes
L-02-02	1.06	8.2	11.6	9.6	13.6	Yes
L-02-03	0.67	0.4	7.9	0.3	5.9	Yes
L-02-04	0.50	0.9	7.1	0.5	3.9	Yes
L-02-05	0.43	0.1	0.7	0.1	0.3	No
L-02-06	0.39	0.4	5.3	0.2	2.3	Yes
R-01-01	2.25	–4.1	14.6	0	36.2	Yes
R-01-02	1.42	–1.2	9.9	0	15.5	Yes
R-01-03	0.81	3.1	7.6	2.8	6.9	Yes
R-01-04	0.59	2.2	5.7	1.4	3.7	No
R-01-05	0.54	0.1	6.8	0.1	4.0	No
R-01-06	0.53	0.1	2.9	0.1	1.7	Yes
R-01-07	0.51	0.6	6.2	0.4	3.5	No
R-01-08	0.49	–0.5	3.1	0	1.7	No
R-01-09	0.46	0.5	2.9	0.2	1.5	No
R-01-10	0.45	–2.2	2.8	0	1.4	No
R-01-11	0.41	0.7	2.2	0.3	1.0	Yes
R-02-01	1.29	–0.6	11.7	0	16.7	No
R-02-02	1.24	0.2	13.2	0.2	18.2	Yes
R-02-03	0.95	0.9	9.8	1.0	10.3	No
R-02-04	0.84	2.2	10.0	2.0	9.3	Yes
R-02-05	0.41	1.7	2.4	0.7	1.1	Yes

**Fig. 3:**
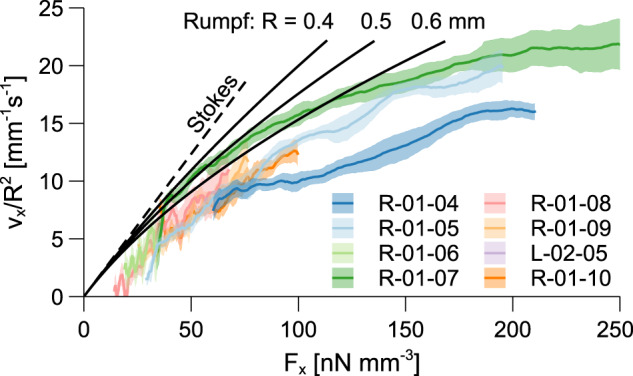
Scaled bubble velocities as a function of the horizontal magnetic force. Results are compared with Eqs. (9) and (10) during the 4.7 s microgravity flight. The legend indicates the bubble ID from Table 2.

Eight bubbles are selected from Table 2 based on the following criteria: (i) bubbles that interact with other bubbles are discarded, (ii) the maximum bubble diameter is 1.2 mm, and (iii) only MilliQ water is considered. In other words, sources of uncertainty are removed by studying small, isolated bubbles in a well-characterized medium. The resulting bubble velocities are scaled with *R*^2^ and plotted in Fig. 3 as a function of the magnetic force in the *x* axis. The ± *σ* error bands from the smoothing velocity filter introduced in sec. “Bubble tracking algorithm” are superposed together with predictions from Eqs. (9) and (10). Since the latter does not scale with *R*^2^, a range of bubble radii are represented. None of the cases under study surpasses the upper-speed limits, validating the application of the proposed magnetic terminal velocity formulations. The measured velocities are, however, significantly smaller than their terminal values. This should not come as a surprise considering the short duration of the experiment, the inhomogeneous magnetic acceleration environment plotted in Fig. 8b, and the wall-induced drag effect described in sec. “Wall–bubble interactions”. The same factors will likely appear in future space applications and should therefore be considered.

### Wall–bubble interactions

The effective drag acting on the bubbles increases by up to two orders of magnitude as they get closer to the wall^71^. This contribution is noticeable already for distances below ~ 10*R*^61^. In close proximity, bubbles will also experience Van der Waals and electrical double layer forces^72^, eventually producing a thin water film between the bubble and the wall. The film drains under the effect of bubble pressure and surface tension, a process that has been successfully modeled by means of force balance-lubrication frameworks^61,73^. The bubble may also invert its curvature close to the surface creating a so-called dimple^74^ and/or bounce back several times before settling^61^.

In the experiments, and as shown in Fig. 8b, the diamagnetic acceleration induced on MilliQ water can reach 0.1–1 m s^−2^ near the magnet. The fundamental processes explored in terrestrial bubbles should be applicable to this experiment by replacing the role of gravity with the diamagnetic force. For instance, the largest bubble from L-01 oscillates several times over the wall of the syringe before being suddenly “absorbed” and starting the film draining process. Such oscillations are relevant for dynamic phase separation approaches and can be studied by means of iterative fluid-magnetic simulations^75,76^ or interface tracking methods^64,77^. The effect of the diamagnetic force in the eigenfrequencies of the bubble depends on the magnetic Bond number at its interface^75^, which is defined as the ratio between magnetic and surface-tension forces. The absorption seems to be related to a change in wettability conditions and can be observed between *t* = 1.5 and *t* = 2.0 s in Fig. 2. It is followed by a slow bubble flattening process where the bubble increases its wall diameter. The same behavior is repeated for all liquids, although the sudden wetting is transformed into a gradual flattening for the LB medium.

**Fig. 4:**
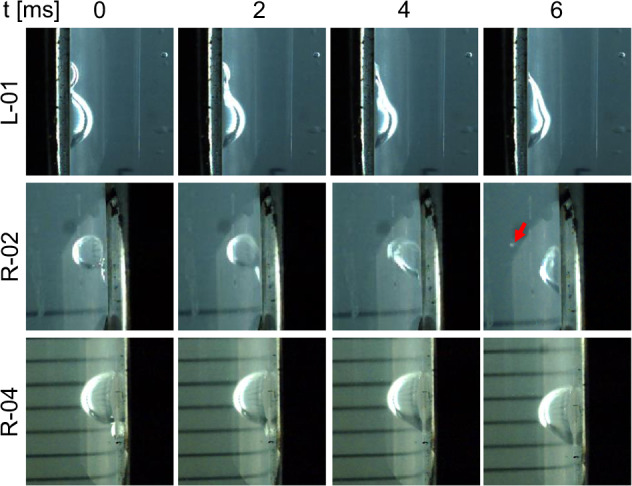
Bubble coalescence events at the wall for the L-01, R-02, and R-04 experiments as a function of time. The red arrow indicates the ejection of a small gas bubble after bubble coalescence.

Of particular technical relevance are the bubble coalescence events reported in Fig. 4 for MilliQ water and LB Broth. The capability to merge several bubbles is key to ensuring a pure gas outcome in future magnetic phase separators. The process is initiated by the thinning of the interface between the two bubbles, which leads to the formation of a neck. The neck expands very fast and starts a damped oscillatory movement in the new bubble that leads to a new equilibrium configuration^78,79^. This cycle is reflected in Fig. 4 and is also observed in bubble–bubble interactions just after injection (see Fig. 2). In some cases, like R-02, a small bubble is ejected due to the violent displacement of the interface. Factors like the concentration of dissolved salts^80,81^ or the bubble collision speed^82^ can determine the likelihood of bubble coalescence, and should therefore be considered in the design of future systems.

### Bubble–bubble interactions

**Fig. 5:**
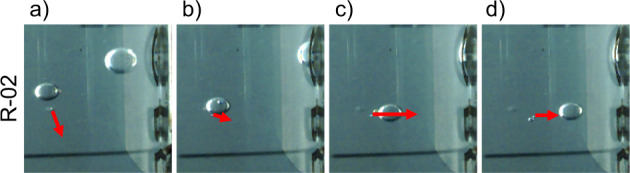
Representative time stamps of a bubble dragging event for the R-02 MilliQ water solution in microgravity. In **a**, the larger bubble approaches the smaller in its path toward the magnet, reaching the point of closest proximity in **b**. In **c**, the smaller bubble is accelerated by the wake of the larger, reducing its speed once the wake moves away in **d**. The red arrow indicates the velocity of the smaller bubble as the larger one drags it.

Equation (9) shows that, at least under the Stokes regime, the terminal velocity of bubbles subject to diamagnetic buoyancy scales with *R*^2^. This characteristic is shared with terrestrial bubbly flows and implies that smaller bubbles will take longer to be separated. For ${{{\rm{Re}}}}\in [20,130]$, a steady wake is however generated behind the bubble with a characteristic length of order *R*^57^. This structure can be used to generate a liquid flow toward the magnet and enhance the collection of small bubbles, as illustrated in Fig. 5 or the stream of bubbles in Fig. 2, R-01. Long-term microgravity experiments are necessary to evaluate this mechanism in a technical setting.

### Relevance to space systems

Among the many space technologies that may benefit from the dia- and paramagnetic phase separation approach and that are briefly listed in sec. “Introduction”, electrolyzers stand out as some of the most interesting applications. Electrolyzers are utilized for oxygen production in the Oxygen Generation Assembly on the International Space Station and suffer from the absence of buoyancy as gas bubble removal from the electrode surface is hindered^1,2,7,8^. The first studies of this phenomenon were carried out in the 1960s within the frame of developing a reliable spacecraft environmental control system for oxygen production^83^. Hitherto, investigations of water electrolysis in drop tower experiments have reported the formation of stable oxygen and hydrogen gas bubble froth layers on the electrode surface with an increasing gas bubble diameter over time. Gas bubbles are observed to adhere to the membrane separating the two half-cells^84–87^. This results in a linear, pH-dependent increase of ohmic resistances with froth layer thickness in both acidic and alkaline electrolytes^84,85^. Generally, larger gas bubbles are formed in reduced gravitational environments, whereas alkaline electrolytes tend to bubble foam. In acid electrolytes, gas bubble coalescence remains a dominant process^84^. These bubble froth layers severely hinder substrate and product transfer to and from the electrode surface and block catalytically active sites on the electrode surfaces^88^. Bubble coalescence is also known to vary considerably in different electrolytes^89^ and to have a strong impact on the overall gas bubble dynamics, which are also influenced by capillary flow and electric forces in microgravity^90^.

A forced water flow can be employed to remove the froth layer from the electrode surface, but the approach has limited efficiency and involves the use of heavy and unreliable liquid circuits^7^. The operation of electrochemical devices with gaseous products or reactants is therefore complicated in reduced gravitation and results in increased complexity, mass, and power consumption. The dia- and paramagnetic phase separation mechanisms illustrated in this paper may thus enable the design of more efficient (photo-)electrolytic cells where bubbles are efficiently removed from the surface of the electrodes and passively collected using magnetic circuits. In combination with well-designed, hydrophilic (electrode) surfaces^91^, the magnetically induced buoyancy approach could provide a key advancement in low-gravity (photo-)electrolysis, boiling, and phase separation systems, among others, which in turn could represent a step-change in enabling human space exploration.

## Methods

### Experimental setup

**Fig. 6:**
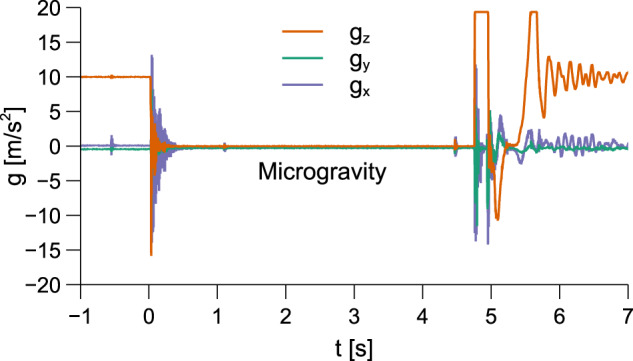
Acceleration profile of the drop capsule measured during the 4.7 s of free fall at the Bremen Drop Tower. Three-dimensional acceleration components are displayed separately, with z denoting the vertical drop direction.

The experimental setup employed in sec. “Results and discussion” is designed to evaluate the dia- and paramagnetic buoyancy effect on three Becton-Dickinson BD Luer-Lok 30 ml syringes that act as sample containers. As previously noted, one syringe is used as a non-magnetic control, while the other two are exposed to the inhomogeneous magnetic field generated by a magnet (see sec. “Magnetic environment”). The experiment is released in a drop capsule from the top of the 120 m ZARM’s drop tower^92^ and experiences ~ 4.7 s of microgravity with maximum gravity residuals of ~ 10^−5^ m s^−2^. The acceleration profile of the experiment is depicted in Fig. 6.

**Fig. 7:**
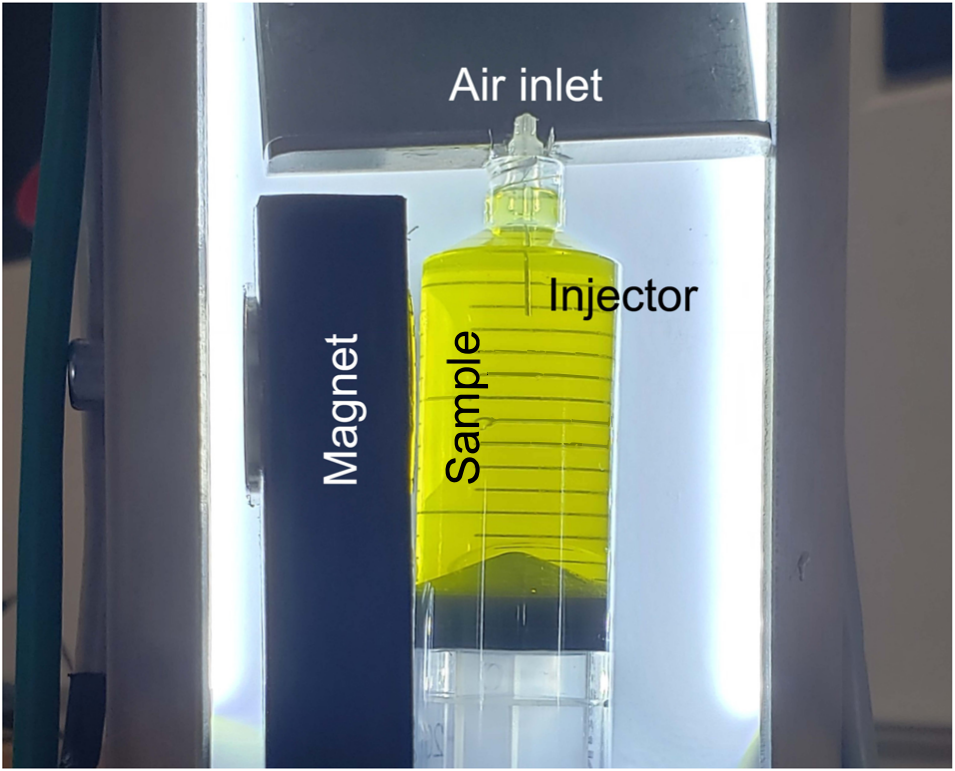
Details of the syringe sample container with the left magnet configuration. Photograph taken by Álvaro Romero-Calvo.

At the beginning of the drop, air bubbles are injected into the syringes using a modified Braun-Sterican 0.3 x 12.0 mm cannula depicted in Fig. 7. The angled tip of the cannula is removed to create a flat air outlet. Its surface is thoroughly cleaned by rinsing with acetone, isopropanol and MilliQ water for 5 s each before hydroxylating the tip for 15 min in a fresh Piranha solution, a 3:1 mixture of sulfuric acid (98%) and hydrogen peroxide (30%)^93^. This procedure is applied to promote the detachment of air bubbles from the tip of the injector. The gas is forced through the cannula by pushing a second syringe connected to the sample container through a silicon tube. A programmable stepper motor is used to simultaneously push the syringes from each sample. In order to minimize air compression effects, part of the tube is filled with water.

The drops are recorded with three Photron MC-2 Fastcam high-speed cameras mounted in front of each container. The cameras are operated at 500 fps with a resolution of 512 × 512 px^2^. This high frame rate requires strong illumination, which is made possible by LED strips that diffusely and homogeneously illuminate the liquid container. The flight sequence is commanded from the Capsule Control System described in ref. ^92^.

### Liquid properties

MilliQ water, LB (Miller) medium, containing 10 g L^−1^ tryptone, 10 g L^−1^ sodium chloride, and 5 g L^−1^ yeast extract, and olive oil possess diamagnetic properties, whereas the Mn^2+^ ion in the 0.5 M MnSO_4_ ⋅ H_2_O solution has five unpaired 3d electrons and is therefore paramagnetic. MilliQ water is well-characterized and exhibits a density *ρ* = 998 kg m^−3^, dynamic viscosity *η* = 1.002 mPa s, surface tension *σ* = 72.75 mN m^−1^, and volume magnetic susceptibility *χ*^vol^ = − 9.022 ⋅ 10^−6^ at 293 K^94^. Therefore, experiments with water are used in sec. “Terminal velocity” to validate analytical formulations. The 0.5M MnSO_4_ ⋅ H_2_O solution is chosen for comparison due to its paramagnetic susceptibility^95^ of ~7.7 ⋅ 10^−5^, while olive oil is characterized by a large dynamic viscosity of ~79 mPa s^96^. Finally, LB Broth is tested due to its widespread application in biological experiments in microgravity and its complex composition^54^.

### Magnetic environment

The magnetic field is induced by a 19.05 mm height, 25.4 mm diameter, 72.4 g N52 neodymium magnet magnetized at 1150 kA m^−1^ and supplied by K&J Magnetics Inc. As shown in Fig. 7, the magnet is mounted on the side of the syringe. Since the magnetic susceptibility of the liquids employed in this experiment is of the order of ± 10^−5^, the magnetic properties of the system can be computed without accounting for the influence of the magnetization field **M** on **H** or the magnetic normal traction term at the liquid-gas interface. This effectively uncouples the fluid-magnetic problem and simplifies the modeling of the system, ultimately enabling the adoption of the external magnetic field **H**_0_ produced by a magnet in a non-polarized environment^53^.

**Fig. 8:**
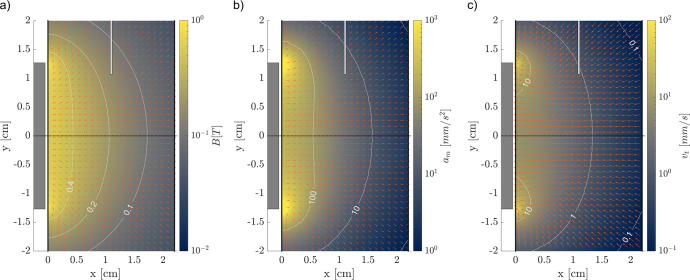
Magnetic environment inside the syringe filled with MilliQ water. The white bar at the top and gray box at the left represent the gas injector and magnet, respectively. The panels represent the **a** magnetic field, **b** diamagnetic acceleration exerted on the liquid, and **c** terminal velocity of a 1 mm diameter air bubble.

The magnetic field, diamagnetic acceleration on deionized water, and terminal velocity of a 1 mm diameter bubble computed from Eq. (10) and *κ* = 2 (${{{\rm{Re}}}} \,<\, 10$) are shown in Fig. 8. Terminal velocities of 1 to 10 mm s^−1^ are obtained between the injector and the magnet, indicating that the bubble reaches the wall of the syringe in a few seconds.

### Bubble tracking algorithm

The characterization of bubbles in transparent liquids is complicated by optical and geometrical challenges that undermine the detection process. Among them, poor illumination conditions, the superposition of different layers of bubbles, or the heterogeneous appearance of the bubble contour^97^. Different methods have been proposed to automatically determine the bubble size distribution of a given image. Optical algorithms are widely extended^98^ and may be classified as geometry or appearance-based^97^. In the former, a circle is fitted to the image edge map using voting techniques such as the Hough Transform^99^ or alternatives like the Concentric Circular Arrangements method^100^. Although geometry-based approaches are particularly susceptible to noise and result in an excessive number of false positives, appearance-based methods require large algorithm training databases^98^.

**Fig. 9:**
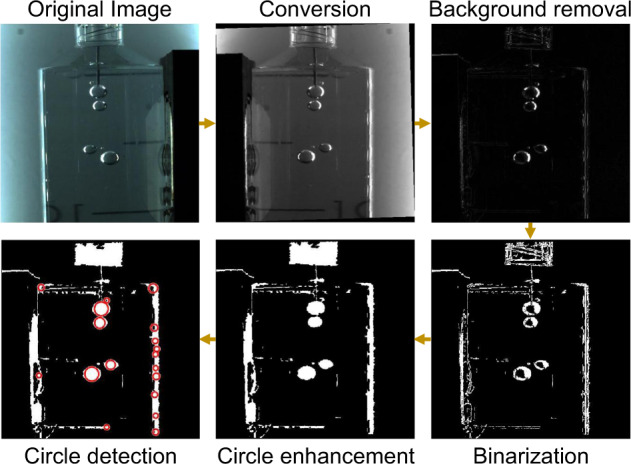
Bubble detection algorithm applied to a representative video frame. The original frame is first converted to gray-scale and flipped if the magnet is located on the right. After background removal, image binarization, and contour closing, bubbles are detected using the Circular Hough Transform. Detected bubbles are shown as red circles in the figure.

A geometry-based algorithm is developed and implemented in Matlab 2021a to track the trajectory of gas bubbles within the syringe. The code is illustrated in Fig. 9 and consists of the following steps:

1. Image conversion: The original video frame is rotated to rectify the camera misalignment and then converted to gray-scale. When the magnet is on the right, the image is flipped to homogenize the comparison between cases.
2. Background removal: The first frame after the start of the drop is subtracted from the current frame to remove background noise.
3. Binarization: The contrast of the image is enhanced before binarizing using Otsu’s method^101^, implemented using Matlab’s imbinarize function. Then, all objects containing less than 5 px are removed with bwareaopen.
4. Circle enhancement: In order to ease the automatic detection of bubbles, a morphological closing is performed with imclose by dilating and eroding the image using a disk shape as structuring element^102^.
5. Circle detection: Finally, circles are detected using the Circular-Hough-Transform-based algorithm implemented in imfindcircles^103,104^. The algorithm is configured with a sensitivity of 0.8 and an edge threshold of 0.2 using a bright object polarity. The curved wall of the syringe elongates the bubble and makes it look elliptical. To correct this visual distortion, a linear transformation is applied in the horizontal direction before detecting the circle and then reversed to compute its actual position.

**Fig. 10:**
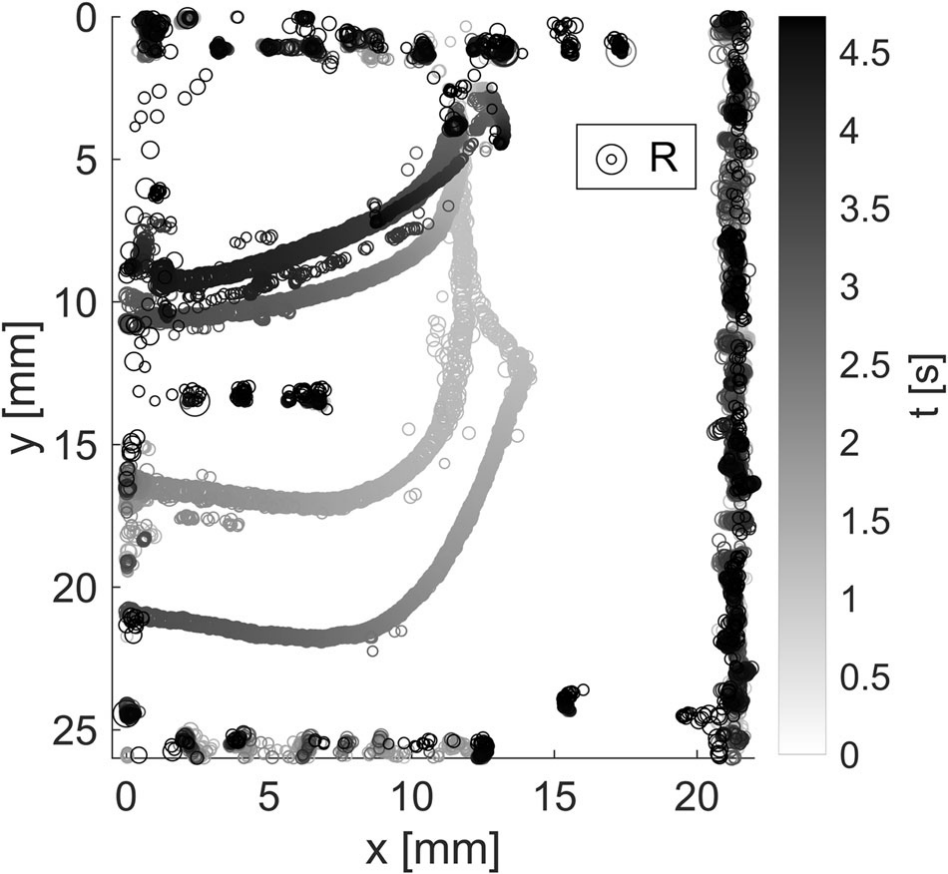
Unfiltered bubble trajectories inside the syringe resulting from the application of the bubble detection algorithm to a 4.7 s drop tower experiment. The color of the markers reflects the time of flight. Their size is scaled between 0.35 and 3.4 mm in radius as indicated by the legend.

The same process is applied to the rest of the video file until all frames are processed. Even though the bubble detection algorithm returns a large number of false positives, the presence of clear structures in the data enables effective post-processing. Figure 10 represents the detected centroid locations as a function of time, with the size of the marker being proportional to the size of the bubble. To reconstruct its trajectory, a manual estimation of the final position is taken by a point tracking algorithm that looks for the closest point within a certain radius in the next frame. Since the initial position of the bubbles is the same, the tracking algorithm is run backward in time. The resulting data are smoothed by applying a moving average filter with a window of 0.2 s. Second-order central finite differences are employed to derive the bubble velocity, which is finally smoothed with the same moving average filter.

### Reporting summary

Further information on research design is available in the Nature Research Reporting Summary linked to this article.

## Supplementary information

Supplementary Materials Information File

Reporting Summary

Movie C-01. Control, drop 1, MilliQ Water

Movie C-02. Control, drop 2, MilliQ Water

Movie C-03. Control, drop 3, 0.5M MnSO_4_·H_2_O(aq)

Movie C-04. Control, drop 4, LB medium

Movie C-05. Control, drop 5, Extra-virgin olive oil

Movie L-06. Left magnet, drop 1, MilliQ Water

Movie L-07. Left magnet, drop 2, MilliQ Water

Movie L-08. Left magnet, drop 3, 0.5M MnSO_4_·H_2_O(aq)

Movie L-09. Left magnet, drop 4, LB medium

Movie L-010. Left magnet, drop 5, Extra-virgin olive oil

Movie R-011. Right magnet, drop 1, MilliQ Water

Movie R-012. Right magnet, drop 2, MilliQ Water

Movie R-013. Right magnet, drop 3, 0.5M MnSO_4_·H_2_O(aq)

Movie R-014. Right magnet, drop 4, LB medium

Movie R-015. Right magnet, drop 5, Extra-virgin olive oil

## Data Availability

All the videos employed in this work are available in the Supplementary Materials.
