# Supplementary material for: Magnetic phase separation in microgravity
Source: NPJ Microgravity. 2022 Aug 8;8:32. doi: 10.1038/s41526-022-00212-9 (PMC9359988; doi:10.1038/s41526-022-00212-9)
Supplement: Supplementary file 1 — Supplementary Materials Information File [file 41526_2022_212_MOESM1_ESM.pdf]

Supplementary Materials for

Magnetic phase separation in microgravity

Álvaro Romero-Calvo, Ömer Akay, Hanspeter Schaub, Katharina Brinkert

Álvaro Romero-Calvo.
E-mail: alvaro.romerocalvo@colorado.edu

This PDF file includes:

Supplementary text
Fig. S1
Table S1
Legends for Movies C-01 to C-05
Legends for Movies L-01 to L-05
Legends for Movies R-01 to R-05

Other supplementary materials for this manuscript include the following:

Movies C-01 to C-05
Movies L-01 to L-05
Movies R-01 to R-05

Supplementary Text

Content. This document lists the 15 videos employed in the main article to analyze different cases of magnetic buoyancy. The videos can be downloaded separately.

Nomenclature. The videos are labeled as N-X, where N stands for "Control" (C), "Left magnet" (L), or "Right magnet" (R) depending on whether a non-magnetic control, left magnet, or right magnet configuration is employed, respectively. An example of each case is shown in Fig. S1. X is the drop identification number (01 to 05). The liquids employed on each drop and their magnetic categories are listed in Table S1.

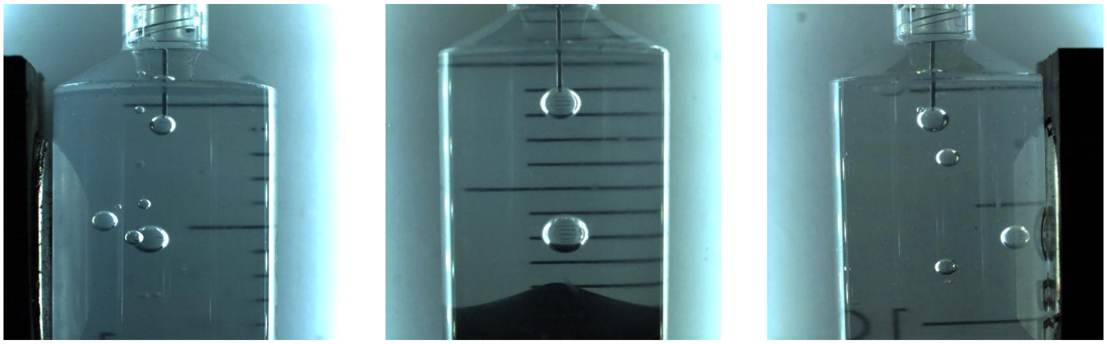

(a) Left magnet (L) (b) Non-magnetic control (C) (c) Right magnet (R)

Fig. S1. Geometrical configurations tested simultaneously in each drop experiment.

Table S1. Liquids employed by the three geometrical configurations in each drop experiment.

ID	Liquid	Classification
01	MilliQ Water	Diamagnetic
02	MilliQ Water	Diamagnetic
03	0.5M $MnSO_4 \cdot H_2O$(aq)	Paramagnetic
04	LB medium	Diamagnetic
05	Extra-virgin olive oil	Diamagnetic

Format. Videos are provided in MP4 format and limited to ~30 Mb each. The original recording is taken at 500 fps and lasts 6 s with a resolution of 512×512 px^2. However, the framerate is reduced to 125 fps to ensure compatibility with common video players.

Video files

Movie C-01. Control, drop 1, MilliQ Water.

Movie C-02. Control, drop 2, MilliQ Water.

Movie C-03. Control, drop 3, 0.5M $MnSO_4 \cdot H_2O$(aq).

Movie C-04. Control, drop 4, LB medium.

Movie C-05. Control, drop 5, Extra-virgin olive oil.

Movie L-06. Left magnet, drop 1, MilliQ Water.

Movie L-07. Left magnet, drop 2, MilliQ Water.

Movie L-08. Left magnet, drop 3, 0.5M $MnSO_4 \cdot H_2O$(aq).

Movie L-09. Left magnet, drop 4, LB medium.

Movie L-010. Left magnet, drop 5, Extra-virgin olive oil.

Movie R-011. Right magnet, drop 1, MilliQ Water.

Movie R-012. Right magnet, drop 2, MilliQ Water.

Movie R-013. Right magnet, drop 3, 0.5M $MnSO_4 \cdot H_2O$(aq).

Movie R-014. Right magnet, drop 4, LB medium.

Movie R-015. Right magnet, drop 5, Extra-virgin olive oil.
